# Bayesian analysis of the association between effective strategies of multimodal nonpharmacological intervention and characteristics of cognitive function in nursing home residents with cognitive impairment: A cross-sectional study

**DOI:** 10.1097/MD.0000000000022154

**Published:** 2020-09-11

**Authors:** Kyosuke Yorozuya, Shingo Yamane, Misako Nobuhisa, Hiroko Owaki, Takeaki Suzuki, Hikaru Okahara, Wataru Iwamori, Hideaki Hanaoka

**Affiliations:** a Rehabilitation unit, Hagijisei Hospital, Hagi, Yamaguchi; b Graduate School of Biomedical and Health Science, Hiroshima University, Hiroshima; c Faculty of Health Sciences, Aino University, Ibaraki, Osaka; d Rehabilitation unit, Geriatric Health Service Facility Jukouen, Ube; e Rehabilitation unit, Geriatric Health Service Facility Shousidou, Hofu; f Rehabilitation unit, Tokuyama Central Hospital Long-Term Care Health Facility, Tokuyama; g Rehabilitation unit, Geriatric Health Service Facility Kourakuen, Yamaguchi; h Aiwa Visiting Nurse Station, Aiwa Co., Ltd., Hiroshima, Japan.

**Keywords:** Bayesian analysis, cognitive function, multimodal, non-pharmacological interventions, nursing home residents with cognitive impairment

## Abstract

The cognitive function of nursing home (NH) residents with cognitive impairment (CI) tends to decline over time. An effective multimodal non-pharmacological intervention (MNPI) strategy is needed to improve the cognitive function of NH residents with CI.

The aim of this study was to clarify the cognitive function characteristics of NH residents with CI in whom a non-pharmacological intervention (NPI) can be implemented, consisting of MNPI using a Bayesian analysis, and to incorporate suggestions to make the MNPI strategy as effective as possible.

This study had a cross-sectional design. The 61 subjects were selected from the residents of 5 NHs, of whom 90.16% were female, and the mean (standard deviation) age was 87.20 ± 6.90. Analyses were performed using a hierarchical Bayesian model, and the global and specific cognitive functions as assessed by the Japanese version of the Neurobehavioral Cognitive Status Examination were the response variables. Three types of NPI (cognitive enhancement NPI, physical NPI, psychological and psychosocial NPI), and activities of daily living (ADL), as assessed by the Barthel index, were the explanatory variables.

Cognitive enhancement NPI was revealed to have no association with any cognitive function. Physical NPI was negatively associated with orientation [OR 0.31 (95% credible interval (95% CI) –2.33, –0.10)], comprehension [OR 0.16 (95% CI –2.78, –0.95)] and naming [OR 0.49 (95% CI –1.47, –0.02)]. Psychological and psychosocial NPI was positively associated with comprehension [OR 3.67 (95% CI 0.52, 2.13)]. Barthel index was positively associated with total Japanese version of the Neurobehavioral Cognitive Status Examination [OR 1.74 (95% CI 0.08, 2.12)], comprehension [OR 3.49 (95% CI 0.45, 4.67)], repetition [OR 10.07 (95% CI 0.53, 9.01)], naming [OR 2.24 (95% CI 0.07, 3.20)], and calculations [OR 18.82 (95% CI 2.71, 9.40)].

The implementation of MNPI should be preceded by cognitive enhancement NPI and physical NPI. Providing ADL enhancing NPI in response to cognitive improvement may be an effective strategy. Providing cognitive enhancement NPI, physical NPI, psychological, and psychosocial NPI, as well as ADL-enhancing NPI at the same time, is also an effective strategy for subjects with mild dementia who are considered to have relatively high cognitive functions.

## 1 Introduction

Many people with cognitive impairment (CI) are eventually admitted to a nursing home (NH),^[1,2]^ and it has been reported that cognitive function tends to decline over time these patients.^[2]^ Furthermore, cognitive deterioration of NH residents with CI may lead to behavioral disorders such as agitation and aggressive behavior.^[3–5]^ CI may also be related to an increased risk of developing a disability.^[6]^ This could incur major physical, mental, and economic burdens for people with CI and their caregivers, and, from a social point of view, increase the cost of medical, and nursing care.^[7,8]^ Improving the cognitive function of NH residents with CI may help solve these problems; however, this remains challenging.

Risk factors for CI include older age, female gender, less education, not being married, a lack of physical, and cognitive activities, and a lack of activities of daily living (ADL) due to disability.^[9,10]^ However, it is difficult for NH residents with CI to intervene in risk factors such as age, gender, educational background, and marital status.

One intervention that has been reported to improve the cognitive function of NH residents with CI is a pharmacological intervention.^[11,12]^ However, pharmacological interventions are not sufficient to improve cognitive function, and, at the same time, have adverse side effects such as weight loss, lower limb cramps, and increased mortality.^[13–17]^ Therefore, the further development of non-pharmacological intervention (NPI), which is expected to have the same beneficial effects as pharmacological intervention, is required.^[18,19]^

In NPIs targeting the cognitive function of people with CI, improvements using a single intervention such as reminiscence, music, and cognitive training, have been studied.^[20–22]^ In recent years, it has been reported that multimodal non-pharmacological intervention (MNPI) can be expected to improve more global and specific cognitive functions than NPI alone.^[23]^

Our systematic review reported that MNPI, which combines exercise, cognitive intervention, and practice in ADL, may be effective in improving global cognitive function, executive function, attention, memory, and structural apraxia in NH residents with CI.^[23]^ However, the characteristics of global and specific cognitive functions of NH residents with CI in whom NPI in the form of MNPI can be carried out remain unknown, and no study has addressed this to date. As such, effective implementation strategies for MNPI, such as the order of provisions and considerations of each NPI according to the state of cognitive function of NH residents with CI, have not yet been studied.

In previous studies on people with CI, analyses using tests and maximum likelihood estimations have primarily been used as conventional statistical methods.^[24,25]^ However, the interpretation of the results of conventional statistical methods is not intuitive, and overfitting is frequently carried out in analyses that combine several types of probability distributions to reflect background information and hypotheses. Therefore, in recent years, Bayesian analyses have garnered attention as a statistical method that is intuitive and easy to interpret, while enabling stable estimation even for complex models.^[26]^

In this study, we clarify the association between each NPI that constitutes MNPI and the characteristics of global and specific cognitive function of NH residents with CI based on the Bayesian analysis predictions. The aim is to discover ways of building a more effective MNPI strategy.

## 2 Methods

This study was approved by the Ethical Committee for Epidemiology of Hiroshima University (E-1587–1). The patients and their caregivers provided informed consent before participation in the study.

This study was structured based on a cross-sectional study design, and subjects were recruited from 5 NHs in Japan. Inclusion criteria were as follows:

1) 65 years of age or older,
2) length of stay in NH of 3 or more months,
3) mini mental state examination-Japanese (MMSE-J) score of 23 or under and clinical dementia rating (CDR) score of 1 or 2 (0, normal; 0.5, questionable; 1, mild dementia; 2, moderate dementia; 3, severe dementia),
4) verbal and written communication is possible and assessment can be performed, and
5) agreement on behalf of the subject and their family to participate in the study.^[27–29]^

Exclusion criteria were as follows:

1) severe behavioral disorders or medical requirements,
2) severe visual or hearing impairment, and
3) refusal to participate in the study.

### 2.1 Data collection

#### 2.1.1 Subject profile

Basic information was obtained from NH medical records, including age, sex, length of stay (months), and diagnosis of dementia. CDR was assessed by NH staff ( Occupational Therapist (OT) or Nurse (Ns)).

#### 2.1.2 Cognitive function

The Japanese version of the Neurobehavioral Cognitive Status Examination (COGNISTAT) was used to assess specific cognitive function.^[30,31]^ The COGNISTAT assesses multi-dimensional cognitive function, and it is constructed of the following 10 subtests (range of raw (standardized) score): orientation (0–12 (0–10)), attention (0–8 (0–10)), comprehension (0–6 (0–10)), repetition (0–12 (1–11)), naming (0–8 (0–10)), constructions (constructional ability; 0–6 (4–11)), memory (0–12 (4–10)), calculations (0–4 (2–10)), similarities (0–8 (6–11)), and judgment (0–6 (6–12)). Standardized scores are used for relative comparison between subtests and for classifying severity levels (9 or more, normal; 8, mild disability; 7, moderate disability; 6 or less, severe disability). The total score ranges from 0 to 82 (23–105) and can also be used as an index of global cognitive function.^[32]^

#### 2.1.3 Content of NPIs

Subjects were surveyed for the frequency, length per session, and type of participation in group NPI as a care practice for the past 3 months. “NPI participation” was defined as participating on average in NPI more than once a week and for more than 30 minutes per session.^[21,23]^ Group NPI as a care practice was mainly conducted by OT, physical therapist (PT), Ns or care worker. Each NPI group consisted of 3 to 20 participants.

In this study, NPIs were classified into 3 types based on previous studies.^[33]^ These included

1) cognitive enhancement NPIs (e.g., calculation tasks, puzzles, and brain training),
2) physical NPI (e.g., gymnastics, stretching, and strength training), and
3) psychological and psychosocial NPI (e.g., singing, Japanese dance, and coloring pictures).

#### 2.1.4 ADL

ADL-enhancing NPI such as practice in ADL were difficult to adequately investigate at the target NHs in this study. Therefore, the Barthel index (BI) assessed by NH staff (OT, PT) was substituted as an indirect index of ADL practice.^[34]^

#### 2.1.5 Statistical analysis

For statistical modeling, Bayesian statistical modeling was used because of its ease of interpretation and stability of analysis in complex models. Unlike conventional statistics, Bayesian statistics consider all parameters as random variables and assume a probability distribution. By combining Bayesian inference and Markov Chain Monte Carlo methods (a random number generator algorithm), the distribution of parameters was estimated as the posterior probability of the obtained data. Stable analysis is possible even for data with a hierarchical structure. It was difficult to obtain a large sample size of NH residents with CI due to severe behavioral disorders, communication difficulties, refusal, and other reasons. Bayesian statistics allow for analyses without relying on *P*-values and can be analyzed stably with a small sample size by using a prior distribution, which was also useful for this study.^[35–37]^

Data for this study were collected from multiple NHs and exhibited a hierarchical structure. Therefore, in modeling, a hierarchical Bayesian model was assumed in which individual differences and group differences were incorporated as random effects for intercepts. The response variables, which were discrete values (raw score of COGNISTAT total and subtests), were associated with a binomial logistic regression model for hierarchical data with random intercepts for both subjects (individual differences) and NHs (group differences). Parameters were estimated using the Markov Chain Monte Carlo methods. The prior distribution was used a half-Cauchy distribution (which is weakly informative prior, and has been recommended).^[38,39]^ Five Markov Chains simulated 25,000 draws and discarded 5000 warm up draws. The appropriateness of the posterior distribution was assessed when Rhat was <1.1.^[26]^

The model selection was based on matching background information, was easy to understand, and was robust. The 3 types NPIs and ADL were the main explanatory variables that were obtained from the background information.^[23,33]^ In addition, age, sex, and length of stay (months) were also considered explanatory variables.^[25]^ The robustness of the model was compared with a simulation using noninformative prior, and it was confirmed whether the result changed significantly or not. The models were verified by assessing a plot representing observed and predicted values. In this study, the emphasis was on whether the provision of each NPI predicted the characteristics of cognitive function, so the models were evaluated as representing “prediction performance”.

Bayesian statistical modeling yields results by an expected a posterior (EAP) and 95% credible intervals (95% CI). EAP is the mean of the posterior distribution. The 95% CI in Bayesian analysis is a posterior interval estimate that has a similar meaning to the 95% confidence interval in conventional statistics and can be interpreted as significant if the value zero is not included.^[36,40]^

In this study, sampled EAP, 95% CIs, and odds ratios were used. The statistical software R (version 3.6.1; R Foundation for Statistical Computing, Vienna, Austria) with the rstan (version 2.19.2) package were used for all statistical analyses.

## 3 Results

In the Bayesian statistical modeling, the posterior distribution approximated a true distribution (Rhat <1.1). The models were also robust. The plots of observed and predicted values were assessed such that the response variables could be approximately predicted by the explanatory variables (Figs. 1–11).

**Figure 1:**
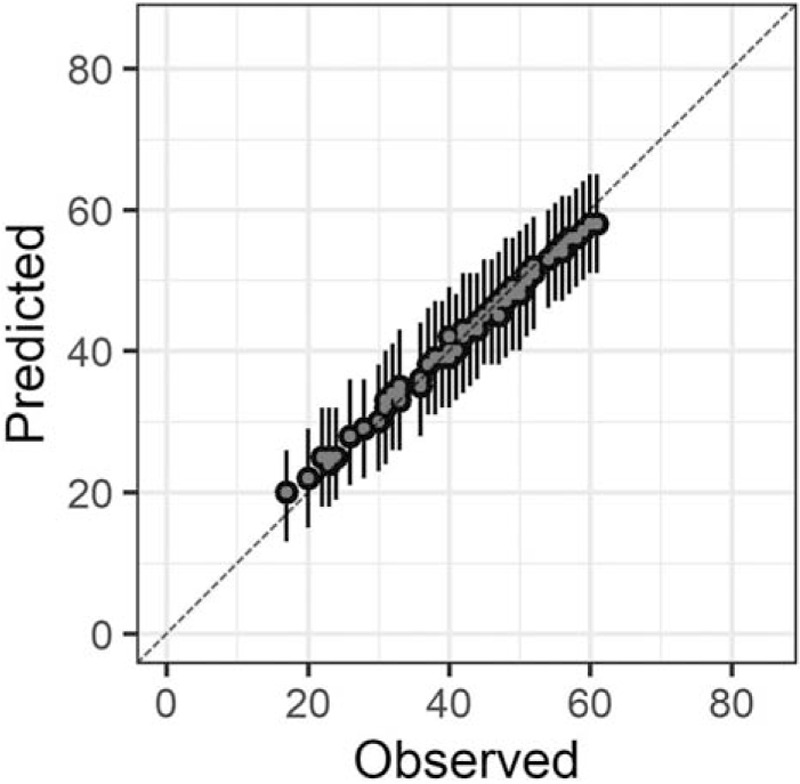
Plots of observed and predicted values. The median predictive distribution and 80% Bayesian prediction intervals are shown. Total Japanese version of the Neurobehavioral Cognitive Status Examination.

**Figure 2:**
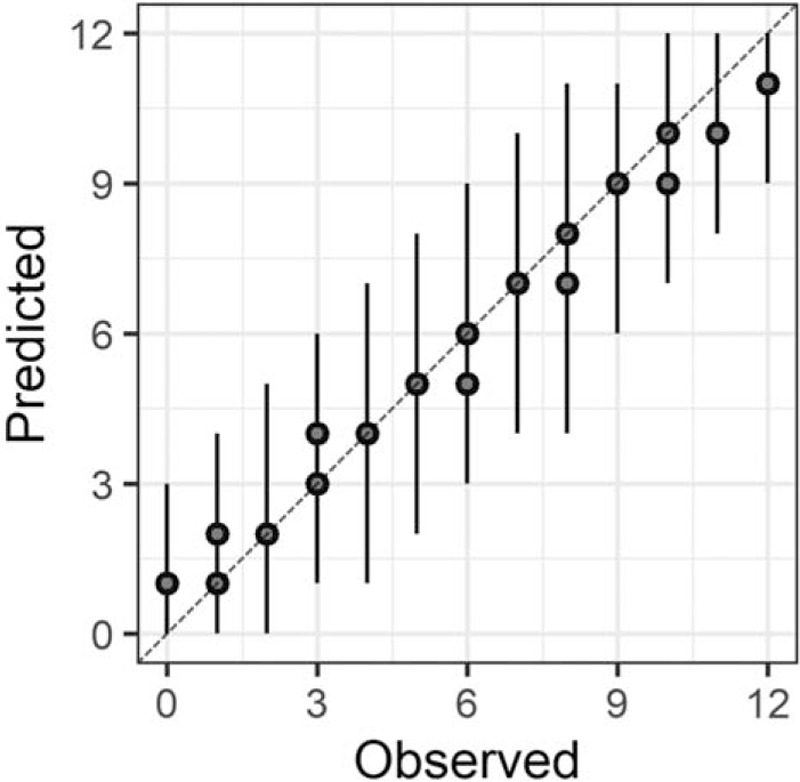
Orientation.

**Figure 3:**
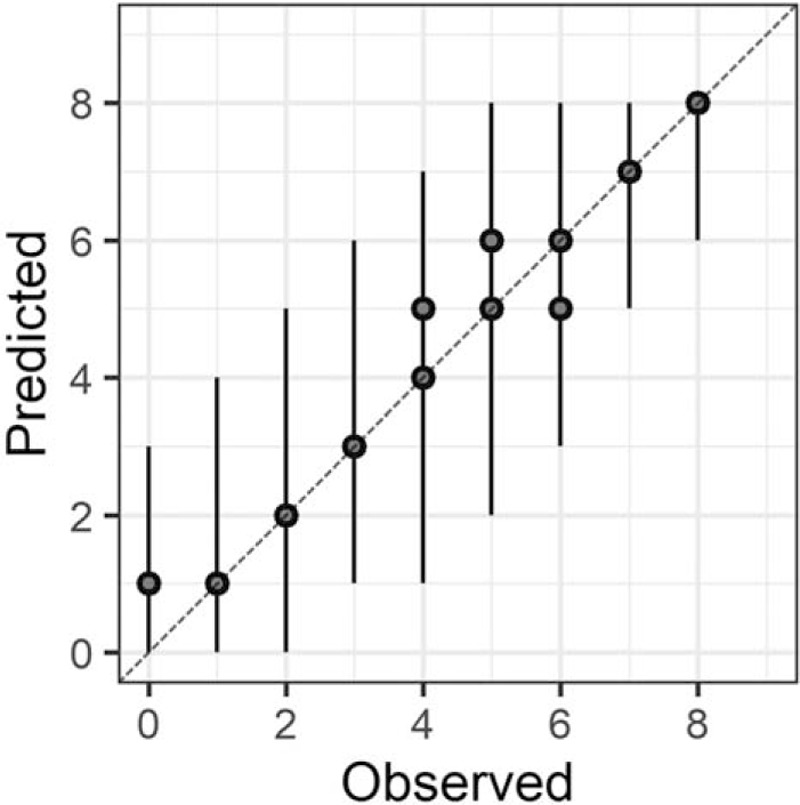
Attention.

**Figure 4:**
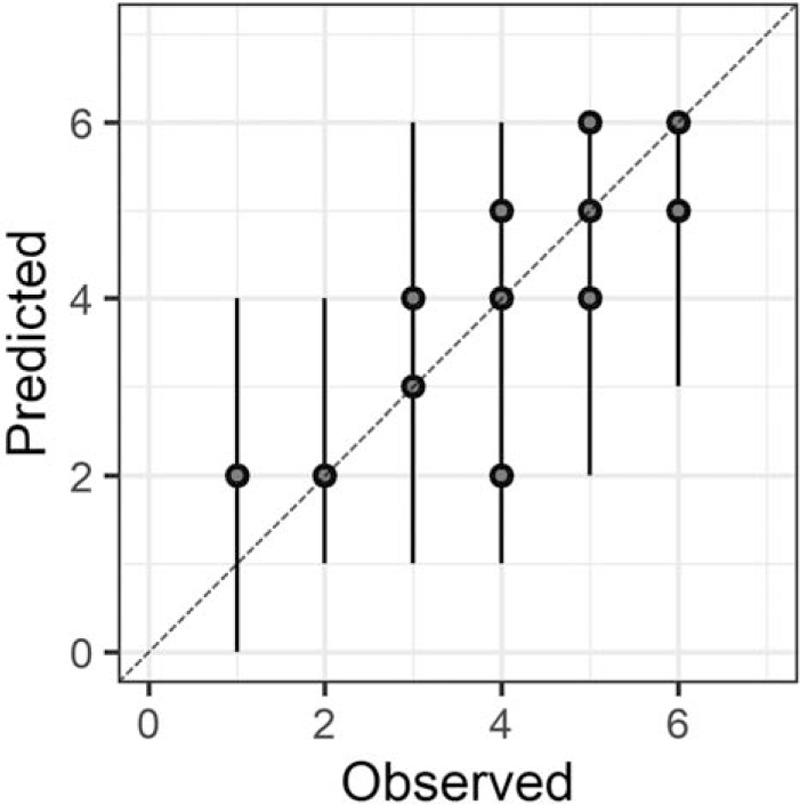
Comprehension.

**Figure 5:**
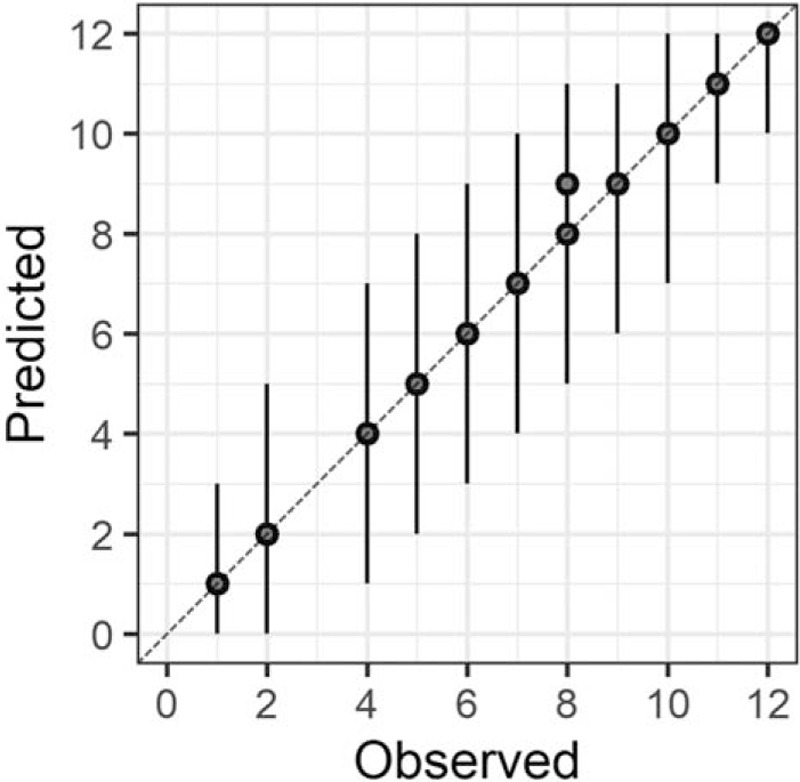
Repetition.

**Figure 6:**
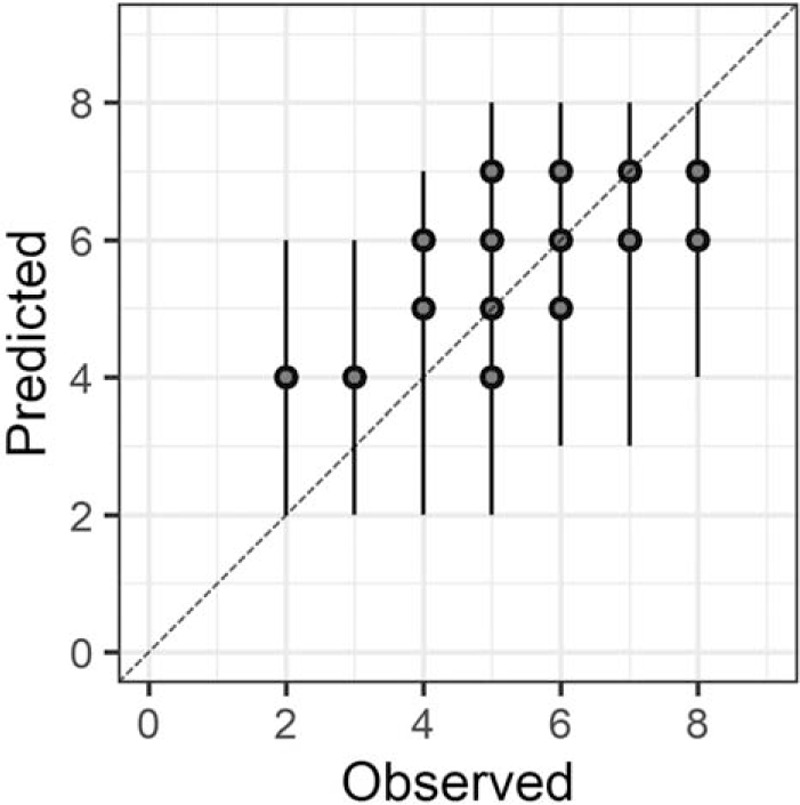
Naming.

**Figure 7:**
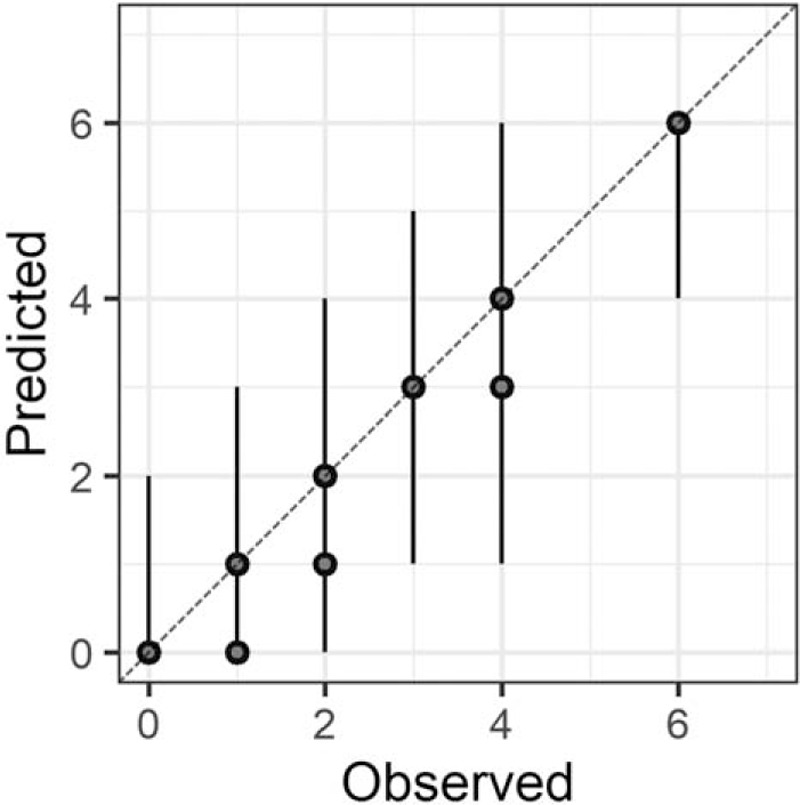
Constructions.

**Figure 8:**
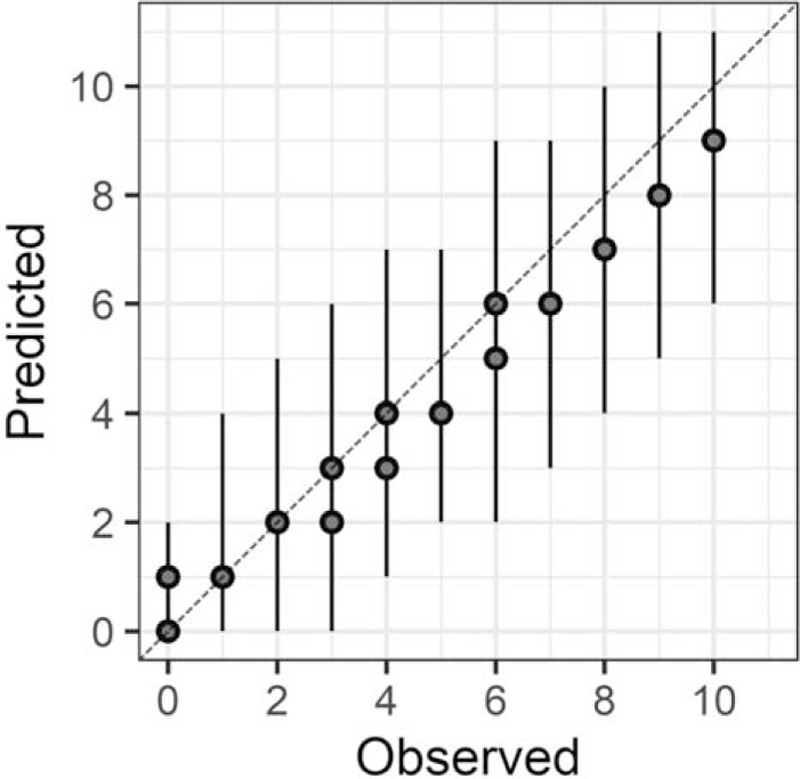
Memory.

**Figure 9:**
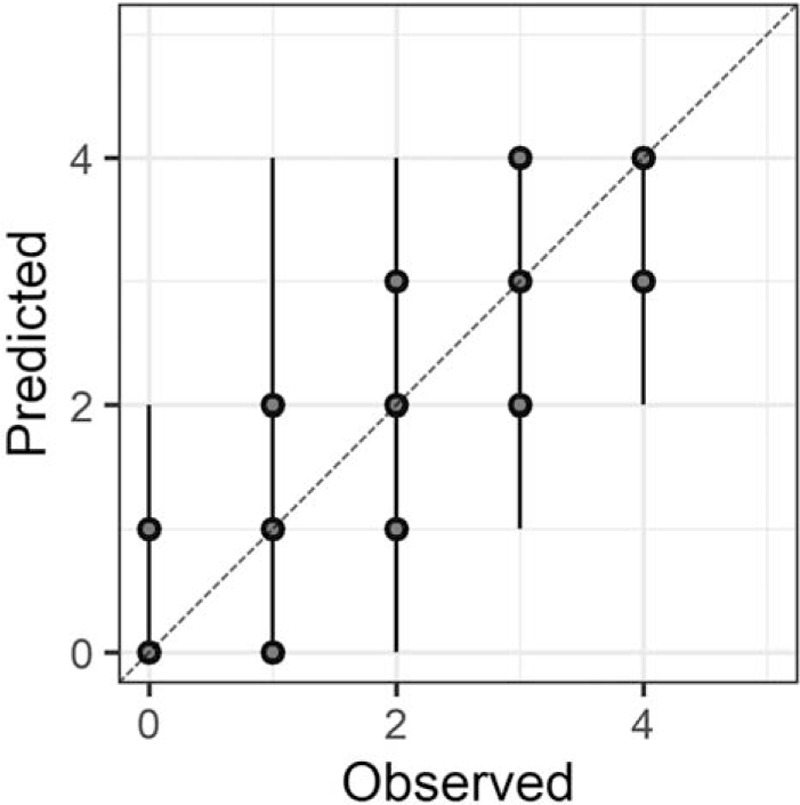
Calculations.

**Figure 10:**
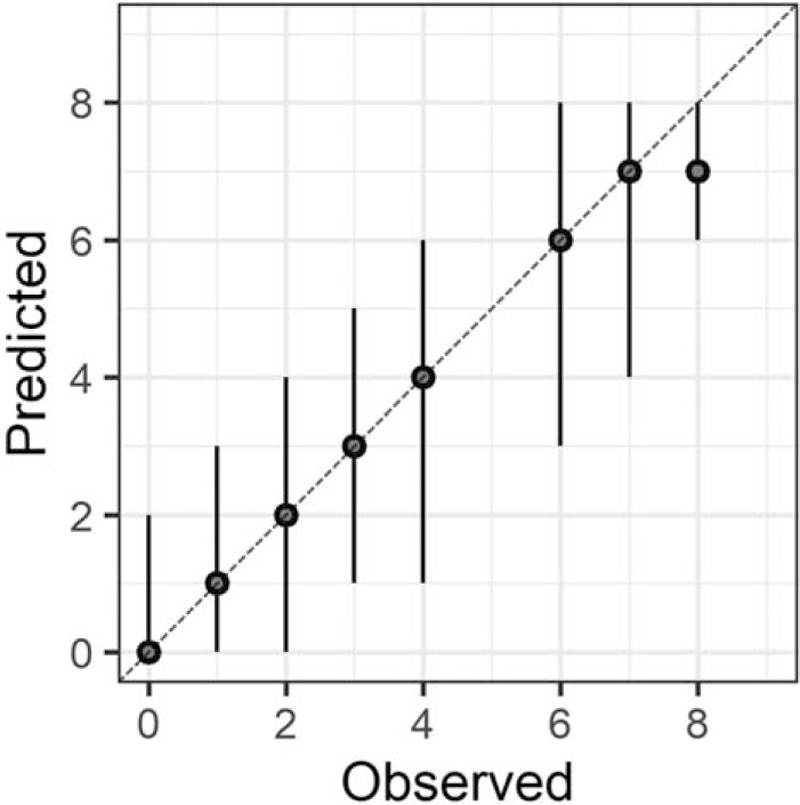
Similarities.

**Figure 11:**
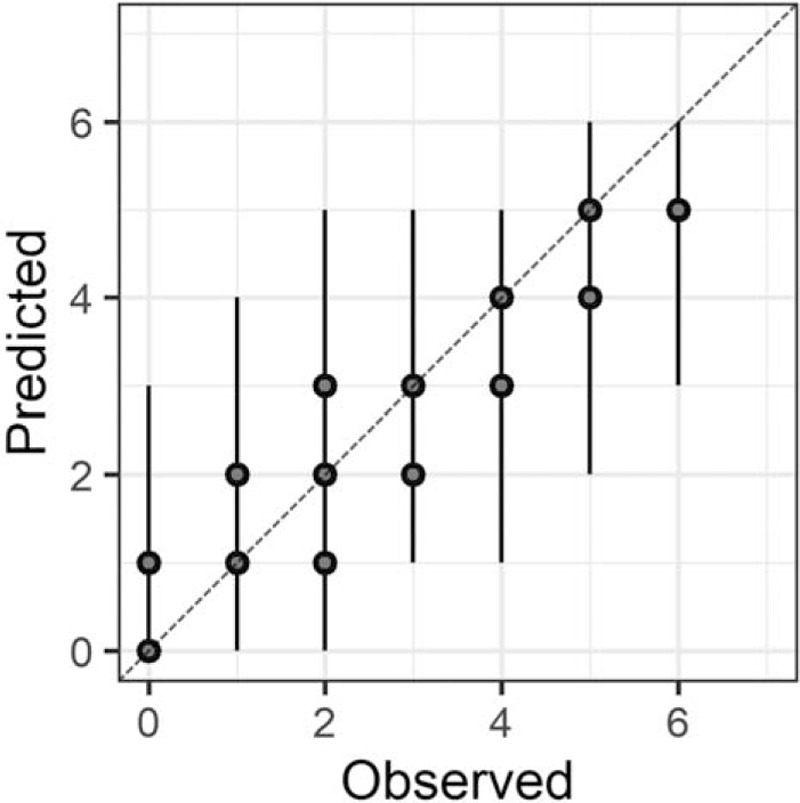
Judgment.

### 3.1 Demographic characteristics

In this study, conducted between May 2019 and February 2020, 61 subjects were selected out of 464 residents. A total of 90.16% were female, the mean [standard deviation (SD)] age was 87.20 (6.90)-years-old, and the median length of stay in the NH was 11 (3–34) months.

Regarding the types of dementia, 11 (18.03%) had Alzheimer disease, 0 (0%) had vascular dementia, 1 (1.64%) had mixed dementia, 18 (29.51%) had dementia (no details), and 31 (50.82%) had no diagnosis. As for CDR, 1scored a 24 (39.34%) and 2 scored a 37 (60.66%). The mean (SD) BI was 70.74 (16.25) (Table 1).

**Table 1:**
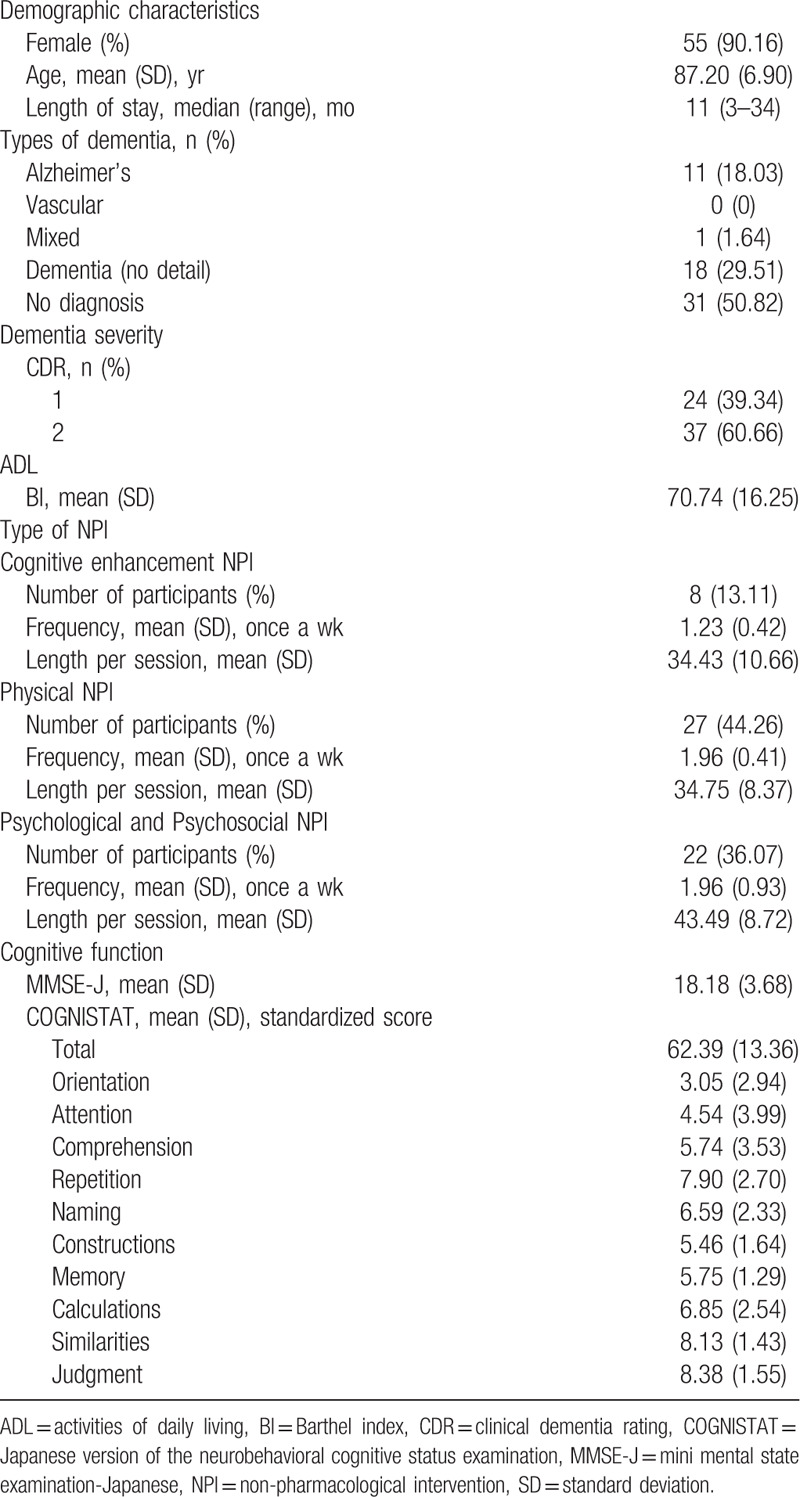
Characteristics of the study population (n = 61).

### 3.2 Cognitive function

The mean (SD) MMSE-J score was 18.18 (3.68). The mean (SD) standardized score of the total COGNISTAT was 62.39 (13.36). The mean (SD) standardized scores of each of the COGNISTAT subtests were as follows: orientation 3.05 (2.94), attention 4.54 (3.99), comprehension 5.74 (3.53), repetition 7.90 (2.70), naming 6.59 (2.33), construction 5.46 (1.64), memory 5.75 (1.29), calculations 6.85 (2.54), similarities 8.13 (1.43), and judgment 8.38 (1.55) (Table 1).

### 3.3 Characteristics by NPI types

The cognitive enhancement NPI values were as follows: the number of participants (%) was 8 (13.11), the mean (SD) frequency was 1.23 (0.42), and the mean (SD) length per session was 34.43 (10.66).

The Physical NPI values were as follows: the number of participants (%) was 27 (44.26), the mean (SD) frequency was 1.96 (0.41), and the mean (SD) length per session was 34.75 (8.37).

The psychological and psychosocial NPI values were as follows: the number of participants (%) was 22 (36.07), the mean (SD) frequency 1.96 (0.93), and the mean (SD) length per session 43.49 (8.72) (Table 1).

### 3.4 Association between Type of NPIs and cognitive function

Cognitive enhancement NPI was not associated with global and specific cognitive function. Physical NPI was associated with orientation [OR 0.31 (95% CI –2.33, –0.10)], comprehension [OR 0.16 (95% CI –2.78, –0.95)], and naming [OR 0.49 (95% CI –1.47, –0.02)]. Psychological and psychosocial NPI was associated with comprehension [OR 3.67 (95% CI 0.52, 2.13)]. BI was associated with total COGNISTAT [OR 1.74 (95% CI 0.08, 2.12)], comprehension [OR 3.49 (95% CI 0.45, 4.67)], repetition [OR 10.07 (95% CI 0.53, 9.01)], naming [OR 2.24 (95% CI 0.07, 3.20)], and calculations [OR 18.82 (95% CI 2.71, 9.40)] (Table 2).

**Table 2:**
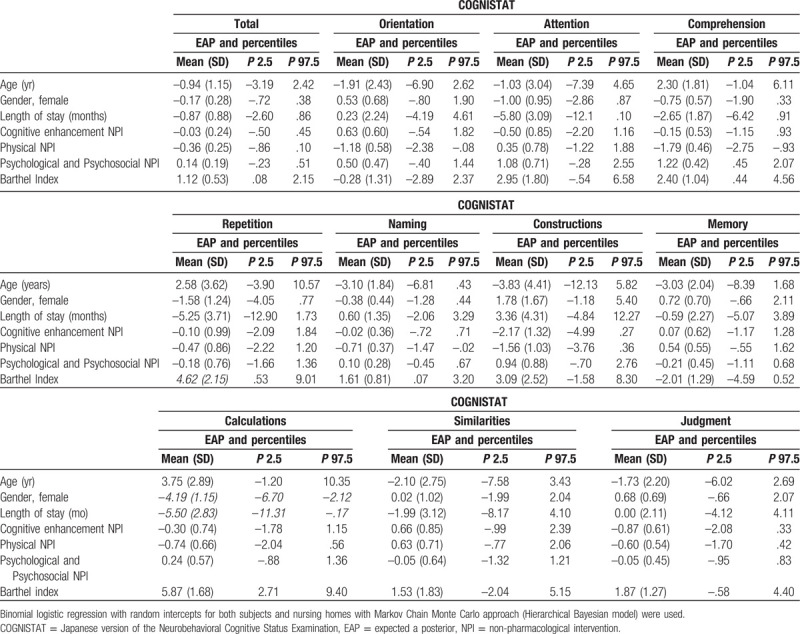
Binomial logistic regression for each variable and Japanese version of the Neurobehavioral Cognitive Status Examination.

## 4 Discussion

### 4.1 Association between NPIs and cognitive function

Cognitive enhancement NPI was revealed to have no association with any cognitive function, and may be provided regardless of the global and specific cognitive functions of NH residents with CI. The cognitive enhancement NPI in this study was based on a group setting, carried out more than once a week and for more than 30 minutes per session. Previous studies have reported that cognitive enhancement NPI improves cognitive function in elderly people in the community and in NHs on a case-by-case basis, when performed approximately 5 times a week for 15 to 20 minute sessions.^[41]^ It has also been reported that cognitive enhancement NPI has the potential to improve cognitive function in patients with mild to moderate CI, and that it can be performed without incurring any burden on participants.^[42]^ The cognitive enhancement NPI may also allow for the difficulty level to be easily adjusted according to the characteristics of the cognitive function.

Those who participated in the physical NPI were predicted to have a higher probability of lower orientation, comprehension, and naming than those who did not. Previous studies have reported that physical activity may be provided more easily if the dementia is severe.^[24]^ Physical NPI has also been reported to improve cognitive function in mild to severe patients with CI.^[43]^ Physical NPI may be easy to provide for NH residents with CI who have particularly low orientation, comprehension, and naming. It has also been reported that physical NPI performed more than once a week and for more than 30 minutes per session may improve the cognitive function of patients with Alzheimer disease.^[44]^ The criteria used for physical NPI design in this study were also similar. These characteristics suggest that physical NPI may be highly useful for NH residents with CI.

Those who participated in the psychological and psychosocial NPI were predicted to have a higher probability of better comprehension than those who did not. Previous studies have reported that activities such as arts and crafts may be offered more easily to individuals with milder dementia.^[24]^ Similarly, as an NPI that requires higher order processing skills, the psychological, and psychosocial NPI used by this study may be more easily delivered to subjects with relatively high cognitive function. Hence, it is necessary to understand how to make it easier for subjects with lower comprehension to participate in psychological and psychosocial NPI. In addition, although psychological and psychosocial NPI was not sufficiently investigated as a component of MNPI in our systematic review, it has been reported that it may be effective for cognitive function in NH residents with CI.^[43,45]^ In the future, it will be necessary to consider MNPI, including psychological and psychosocial NPI.

When BI was high, total COGNISTAT, comprehension, repetition, naming, and calculations were also predicted to be high, with a high probability. In this study, ADL-enhancing NPI such as practice in ADL could not be assessed. It is not directly possible to show whether the ADL-enhancing NPI requires total COGNISTAT, comprehension, repetition, naming, and calculations. However, indirectly, it is speculated that an ADL-enhancing NPI may require a relatively high cognitive function among NH residents with CI. ADL-enhancing NPI may need to take into consideration the participation of subjects with low cognitive function. The implementation of the MNPI as reported in our systematic review may be recommended to be provided first in the form of the cognitive enhancement NPI and then physical NPI, which are considered easier to deliver to NH residents with CI. Second, providing an ADL-enhancing NPI when cognitive function improves may be a more efficient and effective strategy. Providing cognitive enhancement NPI, physical NPI, and ADL-enhancing NPI at the same time is also an effective strategy for subjects with mild dementia that are considered to have relatively high cognitive functions. Furthermore, for subjects with relatively high cognitive function (especially comprehension), adding psychological and psychosocial NPI as a component of MNPI may also be an effective strategy.

The results obtained by the Bayesian analysis can be updated as new data becomes available. It can be assumed that there is no discomfort in daily experience. This leads to the consideration of effective strategies of MNPI based on probabilities and predictions. In the future, the effect of MNPI on global and specific cognitive functions in NH residents with CI needs to be assessed by a longitudinal intervention design.

### 4.2 Limitations

Regarding the diagnosis of dementia, there were many cases of dementia without any details and no diagnosis, and it was difficult to distinguish each type of dementia. No information on educational background was available. The ADL-enhancing NPI could not be assessed directly. This study has potential limitations and a risk of bias which is inherent to cross-sectional studies. There was a great asymmetry in gender distribution (a high proportion of women). The study cannot be said to have external validity, and the results cannot be extrapolated to other populations. There is a lack of available data on several variables which may be potential confounders of the associations between predictors and outcomes (high residual confounding).

The implementation of MNPI should be preceded by cognitive enhancement NPI and physical NPI. Providing ADL-enhancing NPI in response to cognitive improvement may be an effective strategy. Providing cognitive enhancement NPI, physical NPI, psychological and psychosocial NPI, and ADL-enhancing NPI simultaneously is also an effective strategy for subjects with mild dementia that are considered to have relatively high cognitive function. This study will be a beneficial resource for the development of care practices targeted to improving cognitive function in NH residents with CI.

## Acknowledgments

The authors would like to thank Hagijisei Hospital, Hiroshima University, Geriatric Health Service Facility Jukouen, Geriatric Health Service Facility Shousidou, Tokuyama Central Hospital Long-Term Care Health Facility, Geriatric Health Service Facility Kourakuen, and Geriatric Health Service Facility Senogawa for supporting the postgraduate study undertaken by the first author.

## Author contributions

**Data curation:** Kyosuke Yorozuya, Hideaki Hanaoka.

**Formal analysis:** Kyosuke Yorozuya, Hideaki Hanaoka.

**Investigation:** Misako Nobuhisa, Hiroko Owaki, Takeaki Suzuki, Hikaru Okahara, Wataru Iwamori.

**Methodology:** Kyosuke Yorozuya, Shingo Yamane, Hideaki Hanaoka.

**Project administration:** Kyosuke Yorozuya, Shingo Yamane, Hideaki Hanaoka.

**Supervision:** Shingo Yamane, Hideaki Hanaoka.

**Validation:** Hideaki Hanaoka.

**Writing – original draft:** Kyosuke Yorozuya.

**Writing – review & editing:** Hideaki Hanaoka.
